# Supplementary figures and images for: Novel and known signals of selection for fat deposition in domestic sheep breeds from Africa and Eurasia
Source: PLoS One. 2019 Jun 14;14(6):e0209632. doi: 10.1371/journal.pone.0209632 (PMC6568386; doi:10.1371/journal.pone.0209632)

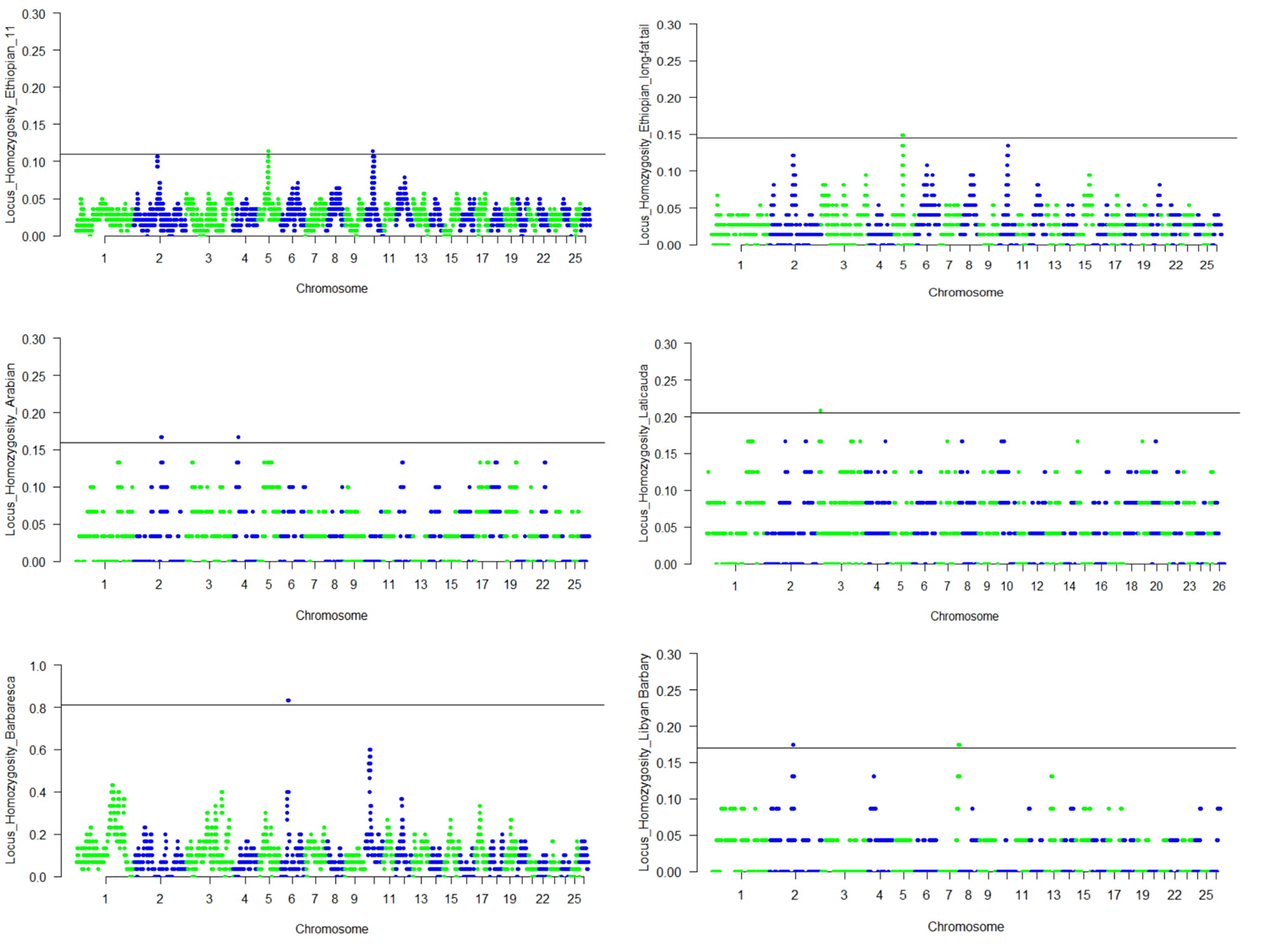

Supplement: S1 Fig — From top to bottom, and from right to left: Ethiopian fat-tail (11 breeds); Ethiopian long fat-tail (6 breeds); Arabian peninsula (Naimi, Najdi, Omani and Huri); Laticauda; Barbaresca; Libyan Barbary. The threshold values (black lines) were based on the criterion of representing the top 0.9999 SNP of the percentile distribution of locus homozygosity values within each group or breed. (TIF) [file pone.0209632.s004.tif]

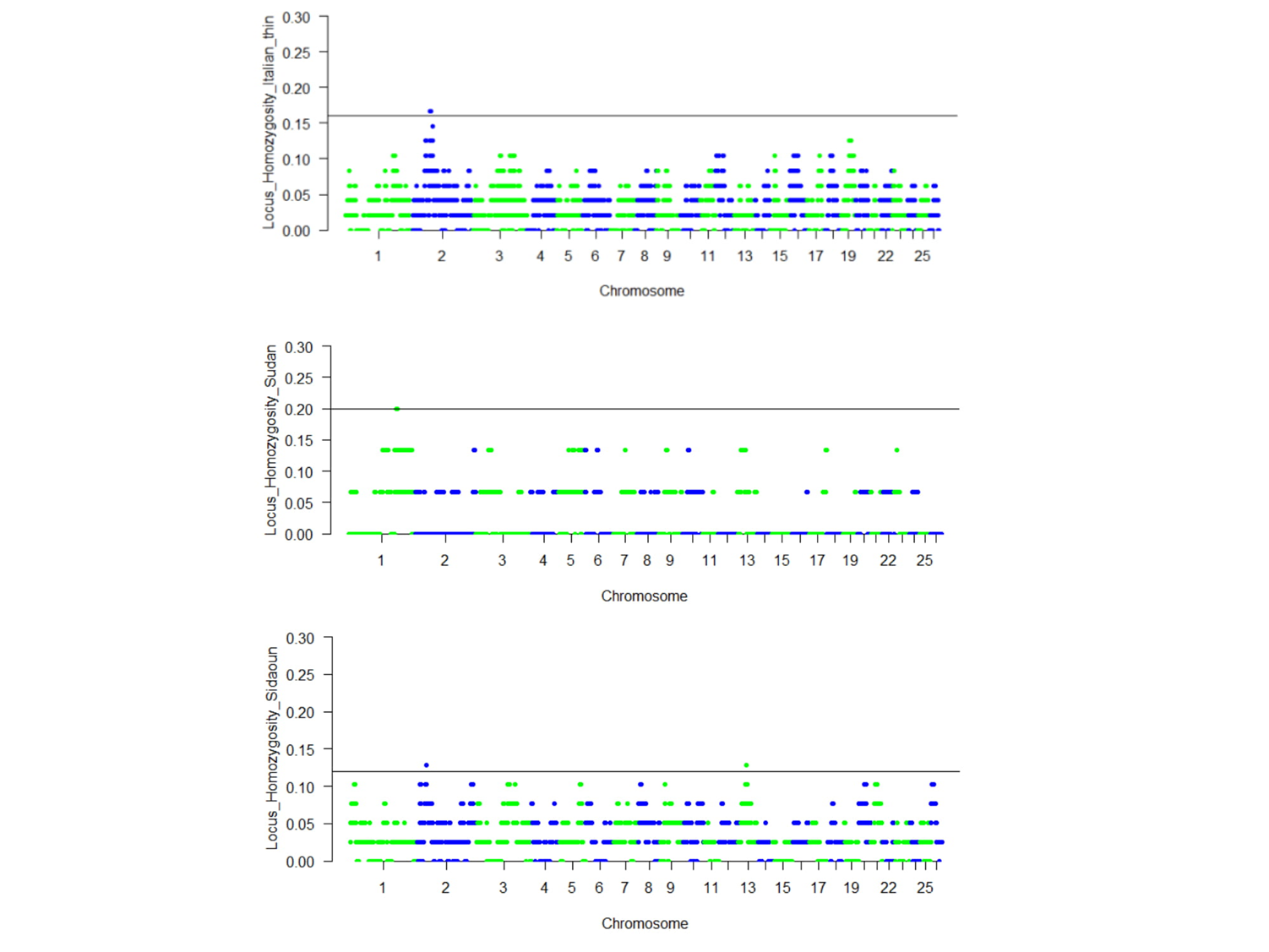

Supplement: S2 Fig — From top to bottom: Italian breeds (Sardinian and Comisana); Sudanese breeds (Hammari and Kabashi); Sidaoun. The threshold values (black lines) were based on the criterion of representing the top 0.9999 SNP of the percentile distribution of locus homozygosity values within each group or breed. (TIF) [file pone.0209632.s005.tif]
